# Alimentary fluoride intake in preschool children

**DOI:** 10.1186/1471-2458-11-768

**Published:** 2011-10-06

**Authors:** Edgar Oganessian, Romana Ivancakova, Erika Lencova, Zdenek Broukal

**Affiliations:** 1 Institute of Dental Research, 1st Faculty of Medicine, Charles University, and General Teaching Hospital, Prague, Czech Republic; 2 Department of Dentistry, Faculty of Medicine, Hradec Kralove and Teaching Hospital, Czech Republic

## Abstract

**Background:**

The knowledge of background alimentary fluoride intake in preschool children is of utmost importance for introducing optimal and safe caries preventive measures for both individuals and communities. The aim of this study was to assess the daily fluoride intake analyzing duplicate samples of food and beverages. An attempt was made to calculate the daily intake of fluoride from food and swallowed toothpaste.

**Methods:**

Daily alimentary fluoride intake was measured in a group of 36 children with an average age of 4.75 years and an average weight of 20.69 kg at baseline, by means of a double plate method. This was repeated after six months. Parents recorded their child's diet over 24 hours and collected duplicated portions of food and beverages received by children during this period. Pooled samples of food and beverages were weighed and solid food samples were homogenized. Fluoride was quantitatively extracted from solid food samples by a microdiffusion method using hexadecyldisiloxane and perchloric acid. The content of fluoride extracted from solid food samples, as well as fluoride in beverages, was measured potentiometrically by means of a fluoride ion selective electrode.

**Results:**

Average daily fluoride intake at baseline was 0.389 (SD 0.054) mg per day. Six months later it was 0.378 (SD 0.084) mg per day which represents 0.020 (SD 0.010) and 0.018 (SD 0.008) mg of fluoride respectively calculated per kg bw/day.

When adding the values of unwanted fluoride intake from the toothpaste shown in the literature (0.17-1.21 mg per day) the estimate of the total daily intake of fluoride amounted to 0.554-1.594 mg/day and recalculated to the child's body weight to 0.027-0.077 mg/kg bw/day.

**Conclusions:**

In the children studied, observed daily fluoride intake reached the threshold for safe fluoride intake. When adding the potential fluoride intake from swallowed toothpaste, alimentary intake reached the optimum range for daily fluoride intake. These results showed that in preschool children, when trying to maximize the benefit of fluoride in caries prevention and to minimize its risk, caution should be exercised when giving advice on the fluoride containing components of child's diet or prescribing fluoride supplements.

## Background

Over the last 15 years, there have been several studies of alimentary fluoride intake in younger pre-school children. Alimentary fluoride intake has been studied in children from 6 months to 10 years in cross-sectional and longitudinal studies in order to determine basal fluoride intake from food sources so that fluoride supplementation in areas with low fluoride content in drinking water can be planned at appropriate doses. The reason why pre-school age children have been selected for the study of fluoride intake is that at this age an early secretory stage of development of the permanent anterior teeth enamel takes place and is very sensitive to increased fluoride intake [1,2]. Fluoride intake has usually been calculated indirectly, based on the records of food and drinks consumed each day and to the known fluoride content of the most frequently consumed types of food and beverages [3-6]. The authors referred to the initial estimate [7,8] that the optimum fluoride intake in children ranged from 0.05 to 0.07 mg/kg of child's weight/day. The range of the optimal fluoride intake has been revised, repeatedly with regard to the possible total amount of fluoride intake from food and swallowed toothpaste [5,7,9].

However, more recent studies have shown that opacities of the permanent anterior teeth might develop even when increased intake of fluoride occurred in children over 3 years [10]. Increased prevalence of opacities in permanent teeth, observed in the U.S.A. and Australia, including areas with low fluoride content in drinking water, has been attributed to the inappropriately high intake of fluoride supplements (tablets, drops) and has led to the decision that the original "optimal" range of daily intake was set at the upper limit [5].

The method of indirect calculation of fluoride intake based on diets and table values of fluoride content in the essential components of child's diet became unsatisfactory and it was replaced by a so-called double-plate method [5]. This method consists of collecting equivalent amount of food and beverages received by a child during the day and then analysing the resulting homogenate to determine the fluoride content directly.

In children aged 3 to 4 years, daily fluoride intake has been found to be in the range from 0.05 to 0.31 mg with an average of 0.15 ± 0.06 mg, calculated to 0.008 ± 0,003 mg/kg bw/day [11,12].

The study of the alimentary fluoride intake in children in the Czech Republic has focused so far on determination of fluoride content in instant milk products, and bottled water. Bottled water for infants is very popular, even in families of preschoolers, for its low content of nitrates, nitrites and sulphates. Still bottled Czech water usually contains 0.04-0.12 ppm F, but among bottled spring water there is one brand with 0.6 ppm F. There is also a bottled table mineral water with a fluoride content exceeding 0.7 ppm, which must be obligatorily labelled as "not suitable for children up to 7 years" [13-15]. Sea food, black tea and other food sources higher in fluoride are rare in the current diet of Czech preschool children.

As to other sources of fluorides, there is no fluoridation of drinking water in the Czech Republic, fluoridated toothpastes for children are available country-wide and fluoride tablets are administered rarely (an estimate in 2008 - fluoride tablets were given to 8% of children aged 0-14 years) and no detailed data are available. The prevalence of dental fluorosis was monitored until 1989 when water fluoridation was stopped and since then no data are available.

The longitudinal study of tooth decay increment, fluoride intake and parental behaviour and attitude regarding oral health of pre-school children has been conducted with following baseline data: n = 300, age 3-4 years, percentage caries free 57.1, dmf mean 1.75 (SD 0.12), 39.25% at need of restorative treatment [16].

### Aim

The aim of this study was to assess the daily alimentary fluoride intake analyzing duplicate samples of food and beverages in a smaller group of individuals recruited from the above larger children's group. An attempt was made to calculate the daily intake of fluoride from food and swallowed toothpaste.

## Methods

The daily alimentary fluoride intake in duplicate samples of food and beverages was assessed in this study.

The Ethics Committees of the 1^st ^Faculty of Medicine in Prague and the Faculty of Medicine in Hradec Kralove approved the study and the parents of children involved in the study signed the appropriate consent form.

The study involved 36 children (18 girls and 18 boys) and their parents (middle to high educational level) from a wider group of children who were taking part in a three-year longitudinal study [16]. None of these children had taken fluoride supplements (tablets) during the study or before. All children were provided with toothpastes of the same brand containing 500 ppm of fluoride and parents were instructed to put only a small pea size of toothpaste on toothbrush. The average age of the children at baseline was 4.75 years. When they were at home all day with their child/children, parents recorded their child's the weight and the volume of food and beverages their children consumed over the course of 24 hours on prepared forms modified, according to local Czech dietary habits, from the forms used in the Iowa fluoride intake study [17]. The parents also collected equal portions of food and beverages consumed by the child. Liquids (water, tea, lemonade, and other beverages) directly measurable potentiometrically for the fluoride content and solid or semi-liquid food (milk, dairy products, soups, pastries, fruit, etc.) not directly measurable for fluoride content were collected separately. The parents were advised, via the study protocol, as to what dietary components of were to be described as "liquids" or "solids" and were asked to estimate amount of food and beverages their child actually received as precisely as possible.

The contents of containers with liquids and solid or semi-solid food were weighted and homogenized in a kitchen blender and the fluoride content was measured subsequently from aliquot sample volumes.

In samples of liquid components TISAB III solution (1:10; v:v) was added for the pH adjustment. Fluoride content was measured directly by potentiometric method using fluoride ion selective electrodes described below.

Aliquot samples of solid and semi-solid food components with an added known volume of deionised water were also homogenized and processed by the quantitative extraction of fluoride by a micro diffusion method according to Taves (1978) [18] as modified by Van Winkle et al. (2005) [10].

A one ml sample of solid food was put in a 10 cm plastic dish followed by 2 ml of 5 M perchloric acid saturated with hexadecyldisiloxane. One ml of 0.1 M sodium hydroxide solution was added into a 3 cm plastic dish as the trapping solution. This dish was then placed on the 10 cm dish with a cover and immediately sealed with vaseline to prevent air leakage and subsequently incubated at 40°C with continuous shaking by rotary motion at 100 rpm for at least 12 hours. One ml of deionised distilled water was added into the trapping solution in the 3 cm dish and the pH was adjusted with TISAB III solution.

The efficiency of the measurements was evaluated using reverse extraction of standard fluoride solution at a concentration of 0.1 and 1 ppm.

The determination of fluoride level was performed potentiometrically using a combined fluoride selective electrode ELIT 8221 (Nico, USA) with a detection limit of 0.02 ppm of fluoride per pH meter InoLab pH/ION 735P (WTW, Germany) whilst stirring continuously in an electromagnetic stirrer. Values in mV were read after stabilization of measured potential. In each sample, the measurement was performed in triplicate. The concentration of fluoride in mg/l was calculated based on calibration values of sodium fluoride solutions at concentrations of 0.02, 0.05, 0.1, 0.2, 0.5, 1.0, 1.5 and 2.0 mg/fluoride per litre.

Determination of daily fluoride intake in children was conducted twice, once at baseline and then again after a six-month interval. In each sample, the measurement of fluoride content was conducted in triplicate and the average values were calculated from the results. When calculating the daily fluoride intake, the following factors were taken into account: child's weight, weight/volume of samples of received food and beverages from duplicate samples and determined fluoride content in formulas. Fluoride content was expressed in mg per 1 kg of consumed food and beverages and in the calculations of daily intake in mg/kg bw/day.

### Validation of the method of quantitative fluoride extraction

To test the method of quantitative fluoride extraction from food, beverages and the mixtures in which the fluoride content cannot be determined by direct electrochemical method, baby formula Sunar Complex Premium (Hero, AS, Czech Republic] an instant milk product containing 0.5 mg of fluoride per 100 g of powder was used instead. Recovered milk using deionised water (13.8 g of powder per 100 ml of water) should contain 0.07 mg of fluoride in 100 ml, thus 0.7 ppm of fluoride. The milk was recovered in the test portion recommended by the manufacturer by deionised water with the fluoride content of 0.00, 0.01, 0.02, 0.06, and 1.00 mg of fluoride per litre. Fluoride was extracted subsequently from the samples of recovered baby formula milk, and its content was determined using the above-mentioned methodology. Results are summarized in Table 1.

**Table 1:** The loss of fluoride in the quantitative extraction conducted during the recovery of Sunar Complex Premium at various fluoride contents in the solvent

Baby formula Sunar Complex Premium (HERO AS, Czech rep.)		Theoretical fluoride content (mg F/l)	Min. (mg F/l)	Max. (mg F/l)	Median (mg F/l)	% of extracted fluoride
Deionized water for recovery (mg F/l)	0.00	0.70	0.52	0.68	0.64	91.43
	0.10	0.80	0.65	0.88	0.77	96.25
	0.20	0.90	0.73	0.88	0.87	96.67
	0.60	1.30	0.84	1.28	1.27	97.69
	1.00	1.70	1.45	1.68	1.67	98.24

Mean						96.06

Validation studies of the fluoride extraction method used in the current study showed that in the expected range of the fluoride content in duplicate food samples, 2 to 4% loss of fluoride in the extraction was acceptable; such conclusion corresponds to the results obtained by the authors of this method and modifications of this method [4,12]. Other authors who used the extraction method also took the potential fluoride loss into account.

The data gathered were processed statistically (STATISTICA 10.0., StatSoft^®^, Czech Rep.) with the calculation of means and standard deviations in respective parameters. For the evaluation of differences between fluoride intake in the first and the second stage of study the paired t test for unequal variance was employed (p < 0.05).

## Results

### Determination of daily alimentary fluoride intake

The observed fluoride content in duplicate samples of liquid and solid food components was, based on the weight of duplicate samples provided by parents. The total fluoride intake of liquid and solid food components was then calculated per 1 kg child's weight per day. The results are shown in Table 2.

**Table 2:** Total and calculated daily fluoride intake

	Child's weight (kg)	Liquids* (kg)	Fluoride in liquids (mg/kg)	Solids** (kg)	Fluoride in solids (mg/kg)	Fluoride from liquids (mg/day)	Fluoride from solids (mg/day)	Total fluoride intake (mg/day)	Fluoride intake in mg/kg bw/day
Baseline									

Mean	19.94	0.568	0.656	0.626	0.026	0.373	0.016	0.389	0.020***
SD	1.68	0.091	0.094	0.083	0.024	0.053	0.008	0.054	0.010

After 6 months									

Mean	21.44	0.612	0.548	0.385	0.112	0.335	0.043	0.378	0.018***
SD	1.5	0.102	0.098	0.083	0.06	48	0,012	0.084	0.008

*Liquids (water, tea, lemonade, other beverages) directly measurable for the fluoride content

** Solids - solid or semi-liquid food (milk, dairy products, soups, pastries, fruit, etc.) not directly measurable

*** Difference in the measured fluoride intake (mg/kg bw/day) between baseline and after 6 months - non significant (0.190)

At baseline the average child's weight was 19.94 kg (SD 1.68). The fluoride content in the liquid food component was on average 0.212 mg/kg (SD 0.091) and in the solid food component it was 0.026 mg/kg (SD 0.024).

When calculating the volume and weight of duplicate samples the average sum of daily intake of fluoride in children was 0.389 (SD 0.054) mg of fluoride per day, which represented 0.020 (SD 0.010) mg/kg bw/day.

The average weight of the children at the second measurement after a six months interval was 21.44 (SD 1.50) kg and analogically calculated daily intake was 0.378 (SD 0.084) mg of fluoride per child. Fluoride intake was calculated per 1 kg of child's weight and was 0.018 (SD 0.008) mg/kg bw/day.

The difference in the daily intake of fluoride calculated per child's weight and day between baseline and after six months of study was not statistically significant (p < 0.190).

Using the values of unwanted fluoride intake from the toothpaste shown in the literature [3,19-23] (0.17-1.21 mg per day) it was possible to estimate how the similar intake from fluoride toothpaste in the summation with the results of this study contributed to the total daily intake and in the calculation of the intake of fluoride per 1 kg bw/day. The estimate of the total daily intake of fluoride amounted to 0.554-1.594 mg/day and recalculated to the child's body weight to 0.027-0.077 mg/kg bw/day (Table 3).

**Table 3:** Estimate of daily accumulation of fluoride intake from food and toothpaste

	Weight of the child *		Fluoride intake in mg/day		Estimated intake of fluoride in mg//kg bw/day
	kg	Total fluoride intake from drinks+food (mg/day)*	Intake from toothpaste**	Total	

Mean	20.69 SD 1.59	0.384 SD 0.069	0.170-1.210	0.554-1.594	0.027-0.077

* Average from two stages of fluoride intake study.

** Data taken from [3,19,21-23].

## Discussion

The method of duplicating food samples (double plate approach) for determination of daily fluoride intake has the advantage of greater accuracy than indirect calculations of intake using diets and table values of the fluoride content in the most frequent components of food and beverages in a child's diet. When tracing fluoride sources in a child's diet, assuming that up to 80% of fluoride in the diet of preschool children comes from drinks [7,14], the authors focused their attention on the fluoride content of bottled water as public water supplies in the centres concerned, where the samples of child's diet were collected, contained 0.08-0.15 ppm fluoride and the provision of bottled water for children is very popular. The bottled table mineral water available locally was richer in fluoride (> 0.7 ppm) and is obligatorily labelled as "not suitable for children up to 7 years". Bottled mineral water is given to children irregularly and rarely [14,15]. In an attempt to collect data on the preference for individual brands of bottled water and other beverages provided by families to preschool children, the authors failed to obtain any generalizable results due to both the broad range and the frequent changes of the brands of bottled water that the children consumed and individual and changing habits among families.

The double plate approach therefore provides better evidence for alimentary fluoride intake than the "basket" approach. Even so, the results of this study should be interpreted with caution as they depended on how thoroughly and accurately the parents duplicated the food and drinks that their child actually received. Another limitation of this study is that the duplicate plate method was conducted for only 24 hours, both at baseline and after six months. Recent studies have suggested that it should be done at least for two consecutive days [24-26] in order to provide more accurate estimates. An attempt was made to reduce that limitation of the daily fluoride intake estimates by collecting food samples on days when their parents were off work and at home.

In the results, the values of daily fluoride intake, in proportion to the child's weight were below the previously recognized "optimal" intake of 0.05 to 0.07 [3,7,9], and reached the lower limit of this range only in their maximum values. It should also be taken into account that the measurements and calculations, did not allow for the alimentary intake of fluoride from swallowed fluoridated toothpaste. In few published studies, this part of the daily fluoride intake was measured and added to fluoride intake from food sources [3,21-23].

The changes in measured values of fluoride intake and other calculations that were found, when comparing the two measurements with a 6-month interval, were not significant (p < 0.190). The average proportion of fluoride intake from liquid food components was 92.2% (at baseline 95.8% and six months later 86.6%). This result is consistent with results of other studies [20,22].

Children aged 4 to 5 years accidentally swallow 30 to 40% of the tooth paste when cleaning teeth [22,27]. However the estimate of alimentary fluoride intake from toothpaste in preschool children must be treated with caution as it is highly variable among children [19,28] due to the amount of toothpaste used, its flavour [29] and other factors.

In this study, parents were instructed that the amount of toothpaste, the children were to use for brushing teeth should be the size of a small pea.

When adding the values of unwanted fluoride intake from the toothpaste shown in the age-matched children in the literature [3,20-23] (0.17-1.21 mg per day) it is possible to estimate that fluoride intake on average would reach the lower limit of the safe range (0.05 to 0.07 mg/kg bw/day). This range is supported by previous studies [3-5]. However, for some children this range could be exceeded.

Given that at present, the topical effect of fluoride in the mouth, whether from toothpaste, rinses or other application forms is considered more effective than fluoride intake through the alimentary route and it is moreover associated with the removal of plaque as cariogenic agent, it is useful to maintain alimentary fluoride intake in preschool children below the safe range of daily intake. Furthermore, it should be strictly individualized. If fluoride supplements (tablets) are considered their doses should take the other intakes of fluoride into consideration [30,31]. In addition, child's food and beverages always contain a certain amount of fluoride, therefore it is necessary to monitor daily intake regularly.

Thus the issue of the alimentary intake of fluoride has two facets relating to its possible contribution to the caries preventive effect of topically applied fluorides and the risk, to enamel maturation. During the period when the enamel of permanent teeth is maturing it is vulnerable even to small over-doses of fluoride. Assuming that alimentary fluoride comes mainly from water or drinks and that part of the absorbed fluoride enters the oral environment via saliva, the intake of drinks occurs several times a day and thus the absorbed fluoride can exert some adjunct effect to topically applied fluorides. On the other hand it has to be born in mind that the apparent advantage of the frequent intake of drinks can contribute to the harmful effect of fluoride over-doses on maturing enamel.

The dynamics of fluoride absorption, whether coming from food or swallowed toothpaste depends on the stomach contents, pH and some ions such as calcium and can have a major impact upon the relative risk of developing fluorosis. The enamel maturation can be endangered both by the long-lasting over-intake of fluoride or, to some extent, by the steep peaks of plasma fluoride due to its quick absorption from empty stomach [1,30].

In summary, this study has demonstrated that alimentary fluoride intake in older preschool children cannot compromise the enamel development of anterior permanent teeth. Even though the drinking pattern of children can differ among families this pattern can be stable within families. Thus it is important to consider the whole dentition when assessing risk benefit and any relative changes to fluoride dose over the whole period of tooth development.

Irrespective of the generally accepted major impact of topical fluoride in caries prevention its systemic intake in preschool children can contribute both to its benefit and to the risk of developing fluorosis. One of the main issues of dental public health is to minimize the risk of fluoride overdose by the fluoride and diet counselling both in individual and community levels.

## Conclusions

In the preschool children studied, observed daily fluoride intake reached the threshold for safe fluoride intake. When the potential fluoride intake from unwanted swallowed toothpaste was added, alimentary intake reached the optimum range for fluoride intake. These results showed that in preschool children, when trying to maximize the benefit of fluoride in caries prevention and to minimize its risk, caution should be exercised when giving advice on the fluoride containing components of child's diet or prescribing fluoride supplements.

## Competing interests

The authors declare that they have no competing interests.

## Authors' contributions

EO participated in the design of the study and carried out all laboratory procedures related to the estimation of fluoride content in food samples. EL and RI prepared instructions for collecting food samples of child's diet and the questionnaire for recording food intake during the sampling periods and gathered samples for laboratory processing. ZB designed the study, supervised laboratory analyses and performed the statistical analysis. All authors conceived the study and helped to draft this manuscript and read and approved the final manuscript.

## Pre-publication history

The pre-publication history for this paper can be accessed here:

http://www.biomedcentral.com/1471-2458/11/768/prepub
